# Transfer learning in hand movement intention detection based on surface electromyography signals

**DOI:** 10.3389/fnins.2022.977328

**Published:** 2022-11-09

**Authors:** Rahil Soroushmojdehi, Sina Javadzadeh, Alessandra Pedrocchi, Marta Gandolla

**Affiliations:** ^1^Nearlab, Department of Electronics Information and Bioengineering, Politecnico di Milano, Milan, Italy; ^2^We-Cobot Laboratory, Politecnico di Milano, Polo Territoriale di Lecco, Milan, Italy; ^3^Department of Mechanical Engineering, Politecnico di Milano, Milan, Italy

**Keywords:** convolutional neural networks, deep learning, prosthetic hand, hand gesture recognition, neural prostheses, surface electromyography, transfer learning, few-shot learning

## Abstract

Over the past several years, electromyography (EMG) signals have been used as a natural interface to interact with computers and machines. Recently, deep learning algorithms such as Convolutional Neural Networks (CNNs) have gained interest for decoding the hand movement intention from EMG signals. However, deep networks require a large dataset to train appropriately. Creating such a database for a single subject could be very time-consuming. In this study, we addressed this issue from two perspectives: (i) we proposed a subject-transfer framework to use the knowledge learned from other subjects to compensate for a target subject’s limited data; (ii) we proposed a task-transfer framework in which the knowledge learned from a set of basic hand movements is used to classify more complex movements, which include a combination of mentioned basic movements. We introduced two CNN-based architectures for hand movement intention detection and a subject-transfer learning approach. Classifiers are tested on the Nearlab dataset, a sEMG hand/wrist movement dataset including 8 movements and 11 subjects, along with their combination, and on open-source hand sEMG dataset “NinaPro DataBase 2 (DB2).” For the Nearlab database, the subject-transfer learning approach improved the average classification accuracy of the proposed deep classifier from 92.60 to 93.30% when classifier was utilizing 10 other subjects’ data *via* our proposed framework. For Ninapro DB2 exercise B (17 hand movement classes), this improvement was from 81.43 to 82.87%. Moreover, three stages of analysis in task-transfer approach proved that it is possible to classify combination hand movements using the knowledge learned from a set of basic hand movements with zero, few samples and few seconds of data from the target movement classes. First stage takes advantage of shared muscle synergies to classify combined movements, while second and third stages take advantage of novel algorithms using few-shot learning and fine-tuning to use samples from target domain to further train the classifier trained on the source database. The use of information learned from basic hand movements improved classification accuracy of combined hand movements by 10%.

## Introduction

Over the past several years, electromyography (EMG) signals have been used as a natural interface to interact with several sorts of external devices (Oskoei and Hu, 2007) such as myo-controlled limb prosthetics (Össur, 2022; Ottobock, 2022; Steeper Group, 2022; Vincent Systems, 2020), exoskeletons (Kiguchi and Hayashi, 2012; Myomo, 2022), rehabilitation devices (Leonardis et al., 2015; Gandolla et al., 2016a), wheelchair (Jang et al., 2016), and speech synthesizers (Niu et al., 2015). Although myo-controlled devices hold great promise, there still exist many challenges to use EMG signals for a smooth control. The first challenge is to detect the exact hand movement intention from the hand’s EMG signal.

Traditionally, surface EMG (sEMG) signals were pre-processed and segmented into windows; then, signal features were calculated over each window and fed to a classifier (Abbaspour et al., 2019). A significant challenge in this approach is choosing the right combination of features. Many researchers have tackled this issue by analyzing different feature combinations and evaluating their performance in terms of accuracy, time efficiency, and robustness (Hudgins et al., 1993; Englehart et al., 1999; Phinyomark et al., 2013; Atzori et al., 2014; Abbaspour et al., 2019). However, discovering the best feature set remains an open problem (Khushaba and Kodagoda, 2012; Phinyomark et al., 2013). Recently, researchers proposed deep learning approaches in order to distinguish hand movement classes using sEMG signals, shifting the methodology from feature engineering to feature learning (Atzori et al., 2016; Park and Lee, 2016; Cote-Allard et al., 2019). Although the approach is different, the goals remain the same: improving accuracy, time efficiency, and robustness of classification. A systematic review on deep learning techniques for sEMG-based hand gesture classification published in 2019 (considering papers published from 2014 to 2019) (Buongiorno et al., 2019) stated that the most widely used deep learning method is Convolutional Neural Network (CNN) (Atzori et al., 2016). In a recent article about classifying sEMG data (Rehman et al., 2018), it was also shown that CNNs show better performance compared to Linear Discriminant Analysis (LDA) with handcrafted features in terms of robustness over time. An important factor when using deep learning algorithms is that obtaining acceptable results is highly dependent on the size of the training database (Cote-Allard et al., 2019), where more data corresponds to better classification accuracy. However, in hand gesture recognition based on sEMG signals, creating a sufficiently large and reliable EMG dataset for each individual is not practical, especially if the number of movement classes is high. One way to approach the mentioned challenge is by utilizing Transfer Learning (TL). To facilitate the training process of deep networks in the targeted domain, transfer learning algorithms take advantage of available labeled data in another similar domain (Bengio, 2012; Yosinski et al., 2014). TL can transfer knowledge between similar domains such as (i) multiple subjects or (ii) multiple tasks. When coming to multiple subjects TL, while it is true that inter-subject variability is always present in multi-user sEMG classification problems, there are also undeniable similarities between users’ data. TL could leverage these similarities using the source model pre-trained on source domain dataset to a new subject-specific model (target model) by adding a small number of labeled person-specific data (Du et al., 2017; Sosin et al., 2018; Cote-Allard et al., 2019; Kim et al., 2020; Tam et al., 2021; Hoshino et al., 2022). When coming to multiple tasks TL, certain hand movements, which include multiple muscle groups and a combination of different functions, referred to as “combined hand movements” (e.g., rotating a key in key hole), can be decomposed to a combination of simpler hand movements, which involve smaller set of muscle groups, referred to as “basic hand movements” (e.g., pinching the key or rotating the wrist). Thus, the patterns in EMG recordings of basic hand movements could be useful for distinguishing combined hand movements. The idea of task-transferability between two different groups of movements was tested by two previous studies (Cote-Allard et al., 2019; Chen et al., 2021). However, none of these studies considered the relation between EMG patterns of two basic gestures and their combined movement. It has been seen in muscle synergy studies that a multiphasic movement can be reconstructed by the combination of different muscle synergies (Weiss and Flanders, 2004; d’Avella and Lacquaniti, 2013). Hence, a sufficiently large database with few basic hand movement classes could be employed to classify simultaneous hand movements in a task-transfer framework. An interesting TL approach introduced for image recognition when the number of training samples of target dataset is limited, is few-shot learning, i.e., the task to learn novel concepts from only a few examples for each category (Fe-Fei et al., 2003; Koch et al., 2015; Qiao et al., 2018). Few-shot learning has recently been implemented in EMG classification (Rahimian et al., 2021) with promising results.

This work aims to explore data transferability in sEMG hand gesture classification and to design algorithms to be used by myo-controlled upper limb devices. To this aim, the contributions of this paper are the following. (i) The development of a new subject-transfer framework to be used when a limited amount of data is available for every single subject. In this regard, the effect of different database sizes for each subject and the number of subjects which we borrow knowledge from, on the performance of the proposed novel TL algorithm is explored. (ii) The investigation of valuable shared information between two different movement groups and the design of a mechanism to take advantage of this shared information. To the best of authors’ knowledge, sEMG data transferability between complex movements and their associated basic movements have not been explored in a TL study using a Deep Leaning approach. It is important to mention that all the proposed classifiers are designed with the consideration of real-time execution. (iii) The introduction of a new sEMG-based hand gesture database referred to as “Nearlab dataset.” The main reasons to create a dedicated sEMG database for this paper was first to add combined movements, suitable for task-transfer experiments, which are not present in other public databases. Second, to include certain sEMG variabilities present in real-life applications. Indeed, one of the sources of EMG variability is the orientation of hand when performing the hand gestures. By considering 3 hand orientations when executing each hand gesture, this variability was accounted for in the proposed database. The proposed approaches are tested on Nearlab dataset and on an open-source sEMG dataset (Ninapro database 2) (Atzori et al., 2014). Nearlab dataset and proposed algorithms are made available (github.com/Rahil-Soroush/Nearlab_sEMG_dataset).

## Data preparation

### Surface EMG datasets

#### Nearlab dataset

We acquired sEMG-based hand/wrist movement dataset to compose the Nearlab Dataset, which we made available to the scientific community, as one of the main contributions of this work. This database includes hand muscles’ electrical activities detected by surface electrodes when performing 8 basic hand movements and 6 combined movements. To the best of our knowledge, this is the only published dataset which includes two sets of basic and combined tasks from same subjects, and it is our hope that it will become a useful tool to compare different task-transfer strategies. The Nearlab dataset comprises sEMG signals of 11 able-body subjects (6 males, age 25 ± 3 years). The only inclusion criterion was the absence of a history of neuromuscular disorders. The ethical research committee of Politecnico di Milano approved the data acquisition protocol on October 16th, 2019. All participants had been briefed about the experiments and gave informed written consent.

**FIGURE 1:**
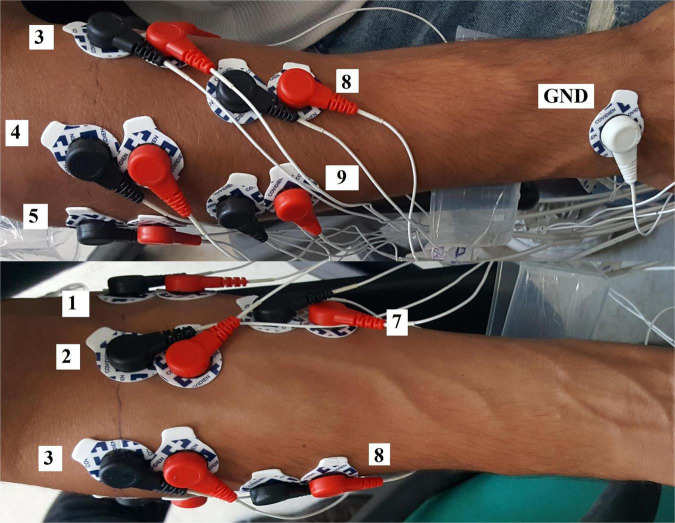
Electrode placement on back (top of the picture) and front (bottom of the picture) of a right-handed subject in the Nearlab dataset. In total, 10 channels were used, 6 around the forearm, and 4 channels 3 cm distal to the previous ones. Electrodes 6 and 10 are not visible in this picture.

Acquisition Setup - SEMG signals have been acquired by 10 differential channels using passive Ag/AgCl electrodes with conductive gel and were sampled at the rate of 2,048 Hz with “Porti” polygraph from TMSi (TMSi, 2022). A Matlab interface was used to visualize and store the acquired signals simultaneously. A trigger input of the acquisition system was utilized to synchronize data with a video containing movement instructions. A micro-controller board (Arduino board) was used to send a pulse to the trigger channel upon receiving the PC’s instruction through a serial connection. The computer would start the video and send the serial command at the same time. The exact positions of bipolar electrodes were determined according to SENIAM guidelines (Figure 1) (Hermens et al., 1999): 6 electrodes pairs around the upper forearm equally spaced along the forearm circumference, and 4 pairs 3 cm distal to the previous electrodes. The reference electrode was placed on the back of the wrist (Hermens et al., 1999). Skin preparation and electrode placement procedures took 20–25 min.

**FIGURE 2:**
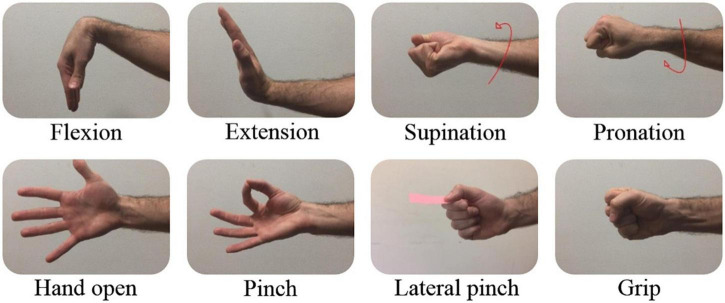
Included movements. 8 Basic hand movements included in the Nearlab dataset.

Experimental Protocol - The experimental protocol was composed of two phases, with instructions presented to subjects through a video cue. Phase 1—each participant was required to perform 15 repetitions of each of the 8 classes of basic movements (Figure 2). In order to consider robustness concerning hand positioning, the total of 15 repetitions per movement accounted for 5 repetitions in each of the following hand postures:

1. Upward starting position, where the palm is faced upward;
2. Sideway starting position, where the palm is faced medially;
3. Downward starting position, where the hand palm is faced to ground.

Movement instructions were presented in random order. Each movement was performed and held for 5 s following 3 s rest and 2 s preparation windows. Phase 2—each participant was required to perform 4 repetitions of each of the 6 classes of combined movements (pronation + pinch, pronation + lateral pinch, pronation + grip, supination + pinch, supination + lateral pinch, supination + grip) with sideway starting position. As before, the order of movements was random. Each movement was performed and held for 7 s following 3 s rest and 2 s preparation windows. Incorrect basic or combined movements were discarded based on operators’ observations at the end of each experimental session, before any processing.

#### Ninapro dataset

Atzori in 2014 (Atzori et al., 2014) published 3 open-source datasets, including several hand and wrist movements, named “Ninapro.” In this study, we used DataBase 2 (DB2) with data from 40 able-body participants. DB2 is collected using 12 active double–differential wireless electrodes with a Delsys Trigno Wireless EMG system (Delsys, 2020), at 2 kHz sample rate. This database includes 3 sets of exercises. The first exercise (called exercise B), which includes 17 basic movements of hand and wrist is used as a reference in this work due to its similarity to movement classes in the Nearlab dataset. Each movement lasts for 5 s and is repeated 6 times. More details on acquisition setup, protocol and movement classes included in exercise B can be found in the original papers (Atzori et al., 2012; Leonardis et al., 2015).

### Preprocessing

#### Nearlab dataset

Nearlab raw data were filtered using a 10–500 Hz band-pass filter (Butterworth 4th order) and a 50 Hz Notch filter (Butterworth 2nd order) (Wang et al., 2013). Filtered data were then divided into labeled movement windows using the video cue time markers. Moreover, windows were further trimmed using a threshold-based onset detection algorithm. This threshold was based on baseline EMG activity during the rest period, obtained by averaging the smoothed rectified signals of 10 channels. In some studies (Gandolla et al., 2016b), the transient part of the signal corresponding to the electromechanical delay is investigated to classify hand movements; however, in this study, the steady-state signal is targeted. Therefore, a small part (100 ms) from the beginning and end of windows were removed to eliminate the transient part of the movement signal (Oskoei and Hu, 2007). The labeled trimmed windows were further segmented to be fed to the network. Considering that for real-time applications, window length plus the processing time to generate classified control commands is suggested to be less than 300 ms (Oskoei and Hu, 2007), a window size of 250 ms (512 samples) was selected. To increase the database size, overlapped windows are extracted from the continuous signal. As suggested in literature (Cote-Allard et al., 2019), we used a sliding window approach with windows of 250 ms (512 samples) and strides of 62.5 ms (128 samples), creating 187.5 ms (384 samples) overlap. Data acquired in phase 1 have been separated into train and test sets. For each hand orientation, 3/5 of each movement’s repetitions were added to the training set, while the remaining were included in the testing set. The data acquired in phase 2 (combined movements) have been kept for testing in task-transfer tests.

#### Ninapro dataset

Ninapro data was already preprocessed by the creators of the database using notch filter (Hampel) to remove power-line interference (50 Hz and harmonics). In the present study the pre-processed data were segmented into windows of 250 ms (512 samples) length and 62.5 ms (128 samples) stride. Repetitions 1, 3, 4, and 6 were included in the training set, and the two remaining repetitions were considered as the test set, as suggested by the introducers of the Ninapro dataset (Atzori et al., 2014, 2016).

## Classification methodology

### Reference classical machine learning algorithms

In order to compare the performance of proposed deep networks, classical classifiers with few selected common features were tested.

#### Feature selection

Based on the available literature on feature selection for sEMG classification (Englehart and Hudgins, 2003; Khushaba and Kodagoda, 2012; Phinyomark et al., 2013) and dataset exploration, 15 time and frequency features were used to create 4 feature sets listed in Table 1. The real-time constraints on feature extraction were considered when selecting the best features. Outlier removal and scaling (using mean and standard deviation) (Vercellis, 2010) were the measures taken to improve the classifier performance.

**TABLE 1:** Selected feature sets and their containing features.

Name	Features	Number of features
Time domain (TD)	MAV, ZC, SSC, WL	4
Improved time domain (ITD)	MAV, ZC, SSC, WL, RMS, IEMG, HP_A, HP_M, HP_C	9
Correlation based (CB)	CC1, ZC, SSC, WL, HP_M, HP_C and SampEn	7
Full feature set (Full)	MAV, ZC, SSC, WL, HP_A, HP_M, HP_C, SampEn, CC1-4, RMS, IEMG, SKEW	15

MAV, Mean Absolute Value; ZC, Zero Crossing; SSC, Slope Sign Change; WL, Waveform Length; RMS, Root Mean Square; IEMG, Integrated EMG; HP_A/HP_M/HP_C, Hjorth Parameters; SampEn, Sample Entropy; CC1-4, Cepstral Coefficients order 4; SKEW, Skewness.

#### Classifiers

We selected (i) K-Nearest Neighbors (KNN) with 40 neighbors and Euclidean distance metric, (ii) Support Vector Machine (SVM) with linear kernel and regularization parameter equal to one, (iii) Multilayer Perceptron (MLP) with 300 neurons in the single hidden layer, tanh as activation function and 0.0001 learning rate and (iv) LDA as reference classifiers, given that they have shown promising results in many studies in sEMG classification, including (Alkan and Günay, 2012; Al-Timemy et al., 2013; Zhai et al., 2016; Abbaspour et al., 2019). The optimized hyperparameters were chosen based on grid search results.

### Deep learning methods

Two baseline CNN s are proposed in this article. Both architectures can be divided into 2 parts. The first part is an inter-connected network of convolutional blocks working as a “feature extractor” and the second part consists of few fully connected layers serving as the “classifier.” The mentioned networks are implemented using Keras (v 2.1.0) (Keras Team, 2022), Python library with Tensorflow (TensorFlow, 2022) backend. Each classifier’s inputs are configured as a 10 × 512 matrix (number of channels × data points in one window). The activation function used in this study is randomized rectified linear unit (RReLU), which was introduced in a recent Kaggle National Data Science Bowl (NDSB) competition (Kaggle, 2022). The following 3 pre-cautions have been taken to prevent over-fitting.

Drop out: Srivastava et al. (2014) presented the dropout technique, in which a group of random neurons with the probability of p (e.g., 0.3) are eliminated from hidden layers. As a result, complex coadaptation of features between neurons can be prevented during training, leading to a reduction of over-fitting.

Batch normalization: Introduced by Ioffe and Szegedy (2015), Batch Normalization (BN) was aimed to solve the need for low learning rate and careful parameter initialization in the training of deep neural networks. It is a type of regularization technique that performs input normalization in each training mini-batch.

Early stopping: This regularization technique monitors the validation error in each update during training. When it reaches a minimum, the learner continues training only for a certain number of iterations and then stops the training. This mentioned number of iterations is referred to as Patience and is set by the user.

#### Deep learning architecture 1: Cnet2D

**FIGURE 3:**
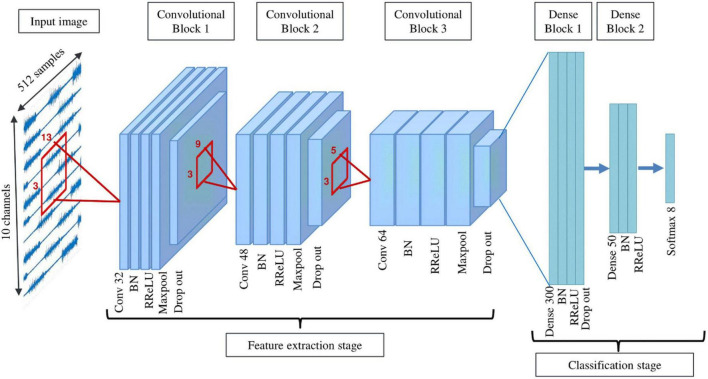
Schematic of Cnet2D and Cnet1D architecture (filter sizes are one dimensional in the case of Cnet1D).

This architecture includes 3 consecutive convolution blocks constructing the feature extractor part, followed by 2 fully connected blocks as the classifier part (Figure 3). Each convolutional block consists of a convolution layer with a 2D filter shape, BN, RReLU activation layer, max-pooling, and dropout. Filter sizes of convolutional layers are (3,13), (3,9), (3,5), respectively. The first fully connected block includes a dense layer, BN, RReLU, and dropout, while the second fully connected block does not include dropout. In the end, a Softmax layer is added to create the output of the classifier. Adam optimizer (Kingma and Ba, 2015) is used as the optimization method. During training, the model with minimum validation loss (20% of training data is randomly selected as validation set) is saved and used for testing. The same approach has been used for all proposed networks. Cnet2D’s classification performance is dependent on electrode positioning due to its 2-dimensional filter shapes. This fact should be considered when applying this network to different databases.

#### Deep learning architecture 2: Cnet1D

The architecture of Cnet1D is similar to that of Cnet2D. However, the filter’s shape is such that it does not exploit the relations between channels in the feature extraction part. Filter sizes of convolutional layers are (1,13), (1,9), (1,5), respectively. Similar to Cnet2D, Adam optimizer is used. Excluding filter sizes, this network’s architecture is the same as Cnet2D depicted in Figure 3. Due to the difference in number of repetitions per movement in the two databases, max-pooling layer parameters are selected differently. These parameters are adjusted based on the validation set (20% of the training set) of each database’s randomly chosen subject. The max-pooling size for Nearlab is (1,4), while for Ninapro is (1,3). Learning parameters of Cnet1D and Cnet2D for Nearlab and Ninapro are reported in Table 2.

**TABLE 2:** Learning parameters of Cnet1D and Cnet2D for Nearlab and Ninapro DB2 datasets.

Parameters	Nearlab	Ninapro DB2
Learning rate	0.001	Reduced from 0.001 to 0.0000005
Epochs	400	600
Early stopping	100	–
Batch size	128	128

### Transfer learning frameworks

Two transfer learning frameworks are defined, which are applied exclusively to previously defined deep neural networks.

#### Transfer learning approach 1: Subject-transfer (PFCnet)

**FIGURE 4:**
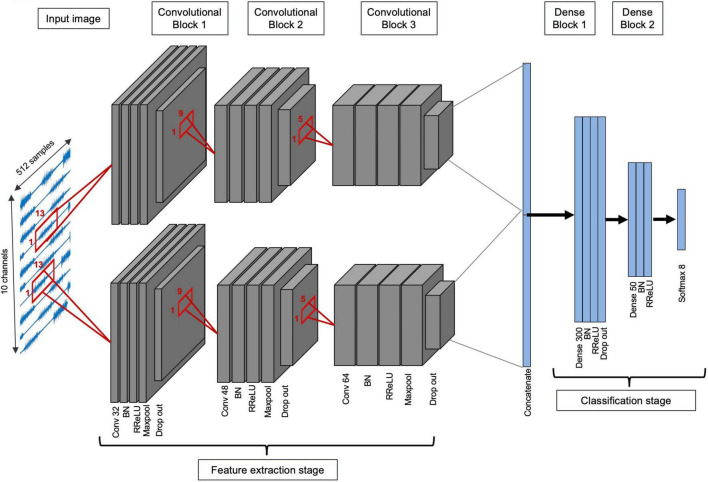
Architecture of the proposed transfer learning method with Cnet1D as base model. The gray layers are pre-trained (top blocks are pre-trained with target subject data, and lower blocks are pre-trained with data from all other subjects), and their parameters will be frozen while the blue layers are trained with the target subject database. Thus, in the process of the proposed transfer learning method, target subject’s data is used two times at two different stages.

In order to apply the subject-transfer framework to Nearlab subjects, one deep network (Cnet1D or Cnet2D) is trained on the target subject’s database (referred to as “Target Network”). Another network (referred to as “Source Network”) is trained on all subjects except the target subject (10 subjects). The features extracted by the mentioned networks are concatenated. Their classifier parts are disregarded, and a new classifier part is added after the feature layer, completing the architecture of “Final Network.” Parameters of the two parallel feature extractors are then frozen (except batch normalization layers), and the classifier part (fully connected layers) is trained using the target subject’s database (with random initialization). Thus, in the process of the proposed transfer learning method, target subject’s data is used two times at two different training stages. This parallel feature architecture is referred to as “PFCnet.” Figure 4 illustrates the final model when Cnet1D is the base deep network. Like Nearlab, in Ninapro, the source network of PFCnet is trained with almost 10 subjects’ EMG data (using more subjects for training the source network was not leading to significant improvement as mentioned in the Results section). To this end, Ninapro subjects are divided into 3 groups: from subject 1 to 15, from 16 to 30, and from 31 to 40. Then the source network is trained within the group containing the target subject. The learning parameters of networks used in PFCnet for Nearlab and Ninapro are mentioned in Table 3. Learning parameters for training target network is the same as parameters mentioned in the previous section (Table 2).

**TABLE 3:** Learning parameters of PFCnet for Nearlab and Ninapro DB2 databases.

Parameters	Nearlab		Ninapro DB2	
	Source	Final	Source	Final
Learning rate	0.003	0.001	0.0003	Reduced from 0.001 to 0.000001
Epochs	100	200	200	400
Early stopping	–	80	80	–
Batch size	128	128	128	128

#### Transfer learning approach 2: Task-transfer

The task-transfer framework aims to transfer knowledge learned from the database including 8 basic movements, to another classification problem containing 6 combined movements. Each combined movement is associated with two basic movements which are always a wrist rotation (pronation or supination) and a hand function (pinch, lateral pinch, or grip). Task-transfer experiments are introduced in three stages. In the first stage, the following question is targeted: is there any valuable shared information to be transferred between source (8 basic movement) and target domain (6 combination movements)? Here we propose a method which uses the classifiers trained on source domain to classify the target domain movement classes with zero labeled target domain samples. This method is relying on the fact that a combined movement can be reconstructed by a combination of muscle synergies used in basic hand movements. An accuracy higher than chance level would imply that source domain data has valuable shared information with target domain data. In the second stage, a few-shot learning approach (Fe-Fei et al., 2003) has been proposed as a transfer learning mechanism to classify the target domain movement classes. Here, only a few samples from each target domain class are needed to implement the proposed few-shot learning algorithm. This stage provides a very general framework, applicable to any other task transfer problem. A significant increase in accuracy from stage one, would mean few-shot learning is a good family of methods to be used in this specific task-transfer problem. In the third and final stage, assuming that at least few seconds of labeled target domain data per class is available, a final framework is proposed. In this framework, the target labeled data is used to train and fine-tune a modified version of classifiers trained on basic movements (source data). While the first stage is designed around the muscle synergy principle, the second and third stages are designed with the following characteristics. (1) Scalable to any choice of movement groups; thus, we did not use ideas that are restricted to our choice of hand gestures. (2) Use minimum amount data from target domain for training.

In the first stage, no training data are used from the combined movement dataset, and the classifiers are only trained with the basic movement dataset. The output layer of the networks is then used to extract more than one intended basic movement. When a combined movement is given to the network, the rotation is first determined by extracting the more probable class between pronation and supination according to associated neurons in the final layer. The hand function is then determined by extracting the most probable class between pinch, lateral pinch, and grip. Thus, six movement classes are recognized based on outputs of 8-neuron final layer. This network is referred to as “Syn0net,” highlighting the fact that combined muscle synergies of basic movements was the main idea behind this network while 0 samples of target domain data was used to train it. An additional experiment is designed to examine the activation function penalization when there is uncertainty in classification. Hence, last layer activation function is replaced with Sigmoid function, trained on the basic movements dataset and tested as described before with combination movements (please see section “Task-transfer experiments” for details). The results from this stage will determine if the information contained in basic movement group is useful by their own to classify the target domain movements (6 combination movement).

In the second stage a 2-branch Siamese network (Koch et al., 2015) is implemented using the classifiers trained on basic hand movement dataset. Each branch of the Siamese network is constructed by removing the last dense layers of the already trained Cnet1D network. For this purpose, the 8-neuron layer and 50-neuron layer are removed revealing the 300- neuron layer as the feature output of each branch. The two branches of Siamese network are then joined with a L1 (absolute difference of each neuron pair) distance layer which provides 300 outputs. Finally, a sigmoid activated neuron is added in the end to generate the similarity score between the two inputs. Same-class input pairs would generate a 0 output while not-same-class input pairs would generate 1 in the output neuron. All network weights of the previously trained networks are locked leaving 301 parameters (300 weights + 1 bias) to be trained. These parameters are then trained with input sample pairs (consisting of same-class and not-same-class pairs) from the source domain data (basic hand movements). To train, 100 epochs are used with decaying learning rate (starting from 0.001 to 0.00005) and batch size of 32. One of the significant advantages of this approach is that, the source domain pairs could be generated in such way that Siamese network could potentially master separating certain classes while not emphasizing on separating other classes. In our specific case we wanted the network to be able to strongly separate hand functions (pinch, lateral pinch and grip) from each other as well as hand rotations (supination and pronation) from each other. At the same time, separating a hand rotation from a hand function would not be as important since all combined movement classes include both a rotation and a hand function. Finally, to generate predictions with this network, the sample to be classified is presented to one of the inputs while a query labeled sample from each class of target domain is presented to the other input. The sample is then classified based on the similarity scores generated for each movement class. Additionally, if few samples from each target class is available a majority voting approach could be used to determine the class. For our Siamese network we have used 5 query samples from each of the 6 combination classes, hence this network is referred to as “Sia5net.”

In the third stage, the network trained with basic movements is modified to adapt to the target domain specification. The last layer which previously had 8 neurons with Sigmoid/Softmax activation function is replaced with a layer with 6-neuron with Sigmoid activation function to meet the number of classes in the target domain (6 combination movement). To train the newly added layer, only 1 repetition of the combination movements (7s of movement per class) is used. Thus, the new layer (which is after a fully connected layer with 50 neurons) with 306 new parameters (50 × 6 = 300 weights and 6 bias parameters) is trained with only approximately 430 samples. It’s important to mention that the parameters from the previous layers are locked (non-trainable). To train, 400 epochs with learning rate 0.01 reduced to 0.000002 and batch size 128 are used. Next, the parameters from the 50-neuron fully connected block before the 6-neuron layer are unlocked. Then 50-neuron block and 6-neuron block are fine-tuned with low learning rate with the same limited training data mentioned before. 15,456 parameters are fine-tuned in this stage. This network is referred to as “FTnet.” To fine-tune, 300 epochs with learning rate 0.0001 and batch size 128 are used. Going further back in the network exposed too many parameters to be trained with small training data and worsened the performance of the network.

### Performance evaluation and statistical analyses

The most common performance measure in a classification problem is “accuracy,” namely the number of correct classifications over the total number of classifications of testing samples. Moreover, it is necessary to ensure that the proposed methods can satisfy the time constraints of real-time applications. In order to do so, one random subject is selected, and the time needed for producing prediction related to each network is calculated by averaging this time over 1,000 repetitions of the procedure. Pairwise Wilcoxon signed-rank test is used to determine if two methods have significant statistical difference in their classification accuracy. Wilcoxon signed-rank test is a non-parametric statistical hypothesis test suitable for a repeated measure design where two different conditions are applied to the same subjects (Scheff, 2016). It is also beneficial when normality in data cannot be assumed (McDonald, 2009), as in our case. For performance comparison of more than two methods (i.e., comparing all classical classifiers and deep networks) simultaneously, a Friedman rank test is employed, followed by a *post hoc* pairwise test with the Wilcoxon signed-rank test. Finally, Holm’s method is applied to significance thresholds (alpha) to control the group comparisons’ family wise error rate.

## Results

### Ninapro dataset

#### Reference classical classifiers

**FIGURE 5:**
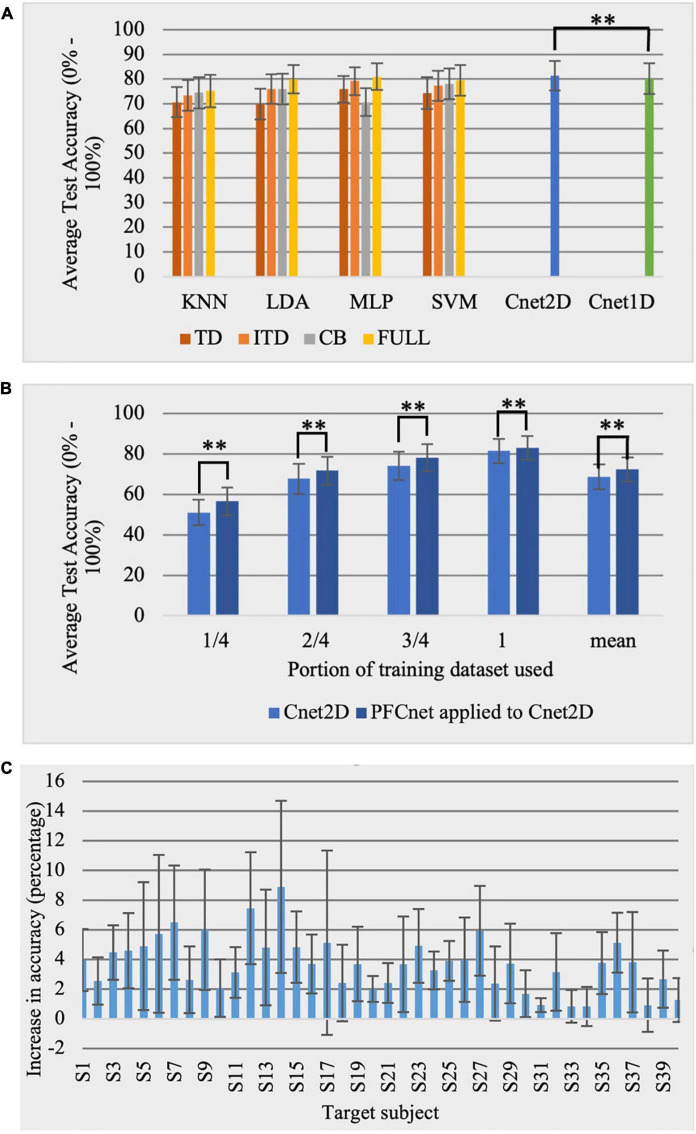
Ninapro dataset results with 17 classes (chance accuracy level = 5.88%). **(A)** Comparison between reference classical classifiers with 4 features sets and the proposed deep networks for Ninapro DB2 database. **(B)** Effect of subject-transfer (PFCnet applied to Cnet2D) for Ninapro DB2 database when training with a limited amount of data (**Corresponds to *p* < 0.01). **(C)** Accuracy improvement when subject-transfer learning is applied to Cnet2D for each Ninapro DB2 subject.

All combinations of 4 selected classical classifiers with 4 feature sets were employed. The average testing classification accuracy and standard deviation over all subjects for Ninapro DB2 exercise B (17 movements) are shown in Figure 5A. In addition, Cnet1D and Cnet2D networks were tested on DB2 subjects, yielding the average accuracy of 80.23 and 81.43%, respectively. All classical classifiers performed the best when using Full feature set.

#### Subject-transfer experiments

The PFCnet method was applied only to Cnet2D, which had higher testing classification accuracy. The need for transfer learning is more evident when the training data is limited. We simulated this situation by training the networks with portions (1/4, 2/4, and 3/4) of the training data of the target subject, removing one entire repetition at a time. The average accuracy over all subjects is shown in Figure 5B. To further investigate the effect of the subject-transfer framework, the improvement in accuracy when transfer learning is applied to Cnet2D is presented in Figure 5C. The reported numbers are the average improvement over different training database sizes (1/4, 2/4, 3/4, and 4/4) for all subjects of Ninapro DB2. Table 4 shows transfer learning applied to Cnet2D (trained on whole training dataset) compared with reference classical classifiers combined with their best feature set. A statistically significant difference was observed using the Friedman test in average accuracy, depending on the classification method (*p* < 0.0001) with PFCnet applied to Cnet2D achieving the highest accuracy. The *p*-values mentioned in Table 4 were acquired by the pairwise Wilcoxon test when PFCnet applied to Cnet2D was compared with other options (e.g., the *p*-value of PFCnet applied to Cnet2D vs. KNN is < 0.00001).

**TABLE 4:** Comparison between classical classifiers, deep network, and proposed transfer learning method, when the whole training dataset is used for training for Ninapro DB2 dataset (*N* = 40) with 17 movement classes (chance accuracy level = 5.88%).

	KNN (Full)	LDA (Full)	MLP (Full)	SVM (Full)	Cnet2D	PFCnet applied to Cnet2D
Average accuracy	75.17	79.95	80.97	79.50	81.43	82.87
Std	6.52	5.73	5.44	6.25	5.94	5.90
*P*-value	0 (<0.00001)	0 (<0.00001)	0 (<0.00001)	0 (<0.00001)	0 (<0.00001)	–
Alpha (Adjusted threshold)	0.01	0.025	0.05	0.0125	0.01667	–

The *p*-values are acquired by the pairwise Wilcoxon test when PFCnet applied to Cnet2D is compared with other options. Holm’s method was applied to significance thresholds to calculate alpha.

### Nearlab dataset

#### Reference classical classifiers

**FIGURE 6:**
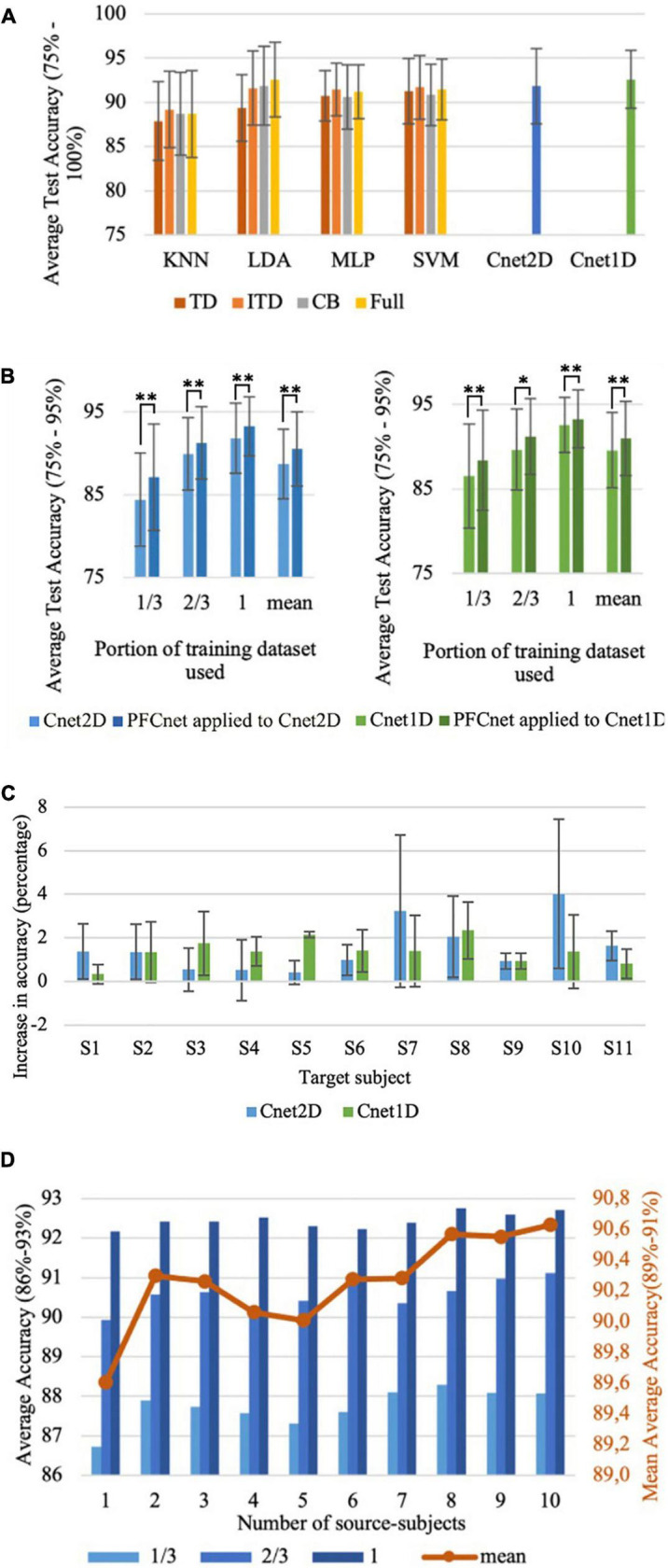
Nearlab dataset results with 8 classes (chance accuracy level = 12.5%). **(A)** Comparison between reference classical classifiers with 4 feature sets and the proposed deep networks for Nearlab database. **(B)** Effect of subject-transfer for Nearlab database when training with different training database sizes. Left is when PFCnet was applied to Cnet2D, and right is the result for applying PFCnet to Cnet1D (*Corresponds to *p* < 0.05 and ^**^Means *p* < 0.01). **(C)** Accuracy improvement when subject-transfer learning is applied to Cnet1D and Cnet2D for each individual of the Nearlab dataset. **(D)** Average accuracy of PFCnet using increasing number of source subjects (selected randomly), across all Nearlab subjects when using 1/3, 2/3, and 3/3 of the target subject’s training data. The red line represents the mean over average accuracies when 1/3, 2/3, and 3/3 are used. In order to better visualize the differences in accuracies, ranges were adjusted to present a zoomed in version of average accuracy in panels **(A,B,D)**.

Combinations of 4 feature sets and 4 classical classifiers were tested on all subjects of the Nearlab database (Figure 6A). Also, the results of the classification accuracy of two proposed deep networks are reported. All classical classifiers except LDA performed best when the ITD feature set was given as input. LDA, combined with the Full feature set, was the best classical classifier for the Nearlab database with 92.55% average accuracy. Cnet1D with 92.6% testing classification accuracy, performed slightly better than LDA and Cnet2D.

#### Subject-transfer experiments

The PFCnet architecture was applied to Cnet1D and Cnet2D. The average testing accuracy over all subjects using 1/3, 2/3, and 3/3 of the target subject training dataset (1/3 portion includes one entire repetition for each movement class starting from all 3 hand orientations and 2/3 include two repetitions) is shown in Figure 6B. The improvement in accuracy when transfer learning was applied to Cnet2D and Cnet1D for all subjects of the Nearlab dataset is presented in Figure 6C. The reported numbers are the average improvement in classification accuracy when 1/3, 2/3, and 3/3 of the target subject’s training data was used. Moreover, classical classifiers with their best feature sets, were trained with different database sizes (1/3, 2/3, 3/3 of the full training data) of the target subject. The comparison between classical classifiers and the proposed transfer learning method applied to Cnet1D are shown in Table 5. Since, according to Figure 6A there was no statistically significant difference between Cnet1D and Cnet2D (*p* = 0.27, pairwise Wilcoxon test), only the results related to Cnet1D, which produced higher accuracy, are reported. In Table 5, a statistically significant difference in average accuracies of the classification methods (KNN, SVM, LDA, MLP, Cnet1D, and PFCnet) was observed using the Friedman test. When 1/3, 2/3, and 3/3 of the training data were considered, the corresponding *p*-values were 0.00022, < 0.00001, 0.00015, respectively. The *p*-values mentioned in Table 5 were acquired by the pairwise Wilcoxon test when PFCnet applied to Cnet1D was compared with other options. To analyze the effect of number of subjects involved in the subject transfer framework, the proposed TL algorithm was re-implemented using increasing number of source-subjects. Figure 6D is displaying the performance of PFCnet when used with 1–10 source-subjects (selected randomly). The accuracies are averaged among all Nearlab subjects selected as target subject. This analysis is done with a variety of training data sizes including 1/3, 2/3 and 3/3 of the available target subject’s training data and reported in Figure 6D. Classifiers’ performance in terms of the time needed to produce a prediction are 4.81, 4.97, and 4.21 ms for Cnet1D, Cnet2D, and PFCnet, respectively. The GPU used for classifications was NVIDIA Tesla P100-PCIE-16GB. It can be inferred that the transfer learning method provides the sample prediction fast enough to meet the time requirements of online applications.

**TABLE 5:** Comparison between classical classifiers and proposed transfer learning method combined with Cnet1D, when different sizes of target’s training subset are used for training for Nearlab dataset (*N* = 11) with 8 movement classes (chance accuracy level = 12.5%).

Portion of the training data		KNN (ITD)	LDA (Full)	MLP (ITD)	SVM (ITD)	Cnet1D	PFCnet applied to Cnet1D
1/3	Average accuracy	82.76	86.42	84.90	85.88	86.55	88.43
	Std	6.27	7.11	6.018	6.18	6.18	5.95
	*P*-value	0 (0.00335)	0 (0.03666)	0 (0.01637)	0 (0.00992)	0 (0.00335)	–
	Alpha (adjusted threshold)	0.0125	0.05	0.025	0.01667	0.01	–
2/3	Average accuracy	84.55	87.74	86.68	86.73	89.68	91.23
	Std	6.21	7.10	5.70	5.92	4.77	4.47
	*P*-value	0 (0.00335)	0 (0.00335)	0 (0.00444)	0 (0.00333)	0 (0.01279)	–
	Alpha (adjusted threshold)	0.0125	0.01667	0.025	0.01	0.05	–
3/3	Average accuracy	89.20	92.55	91.45	91.72	92.60	93.30
	Std	4.35	4.23	2.97	3.60	3.26	3.41
	*P*-value	0 (0.00585)	1	0 (0.00764)	0 (0.00334)	0 (0.00992)	–
	Alpha (adjusted threshold)	0.0125	0.05	0.01667	0.01	0.025	–

The *p*-values are acquired by the pairwise Wilcoxon test when PFCnet applied to Cnet1D is compared with other options. Holm’s method was applied to significance thresholds to calculate alpha.

#### Task-transfer experiments

**FIGURE 7:**
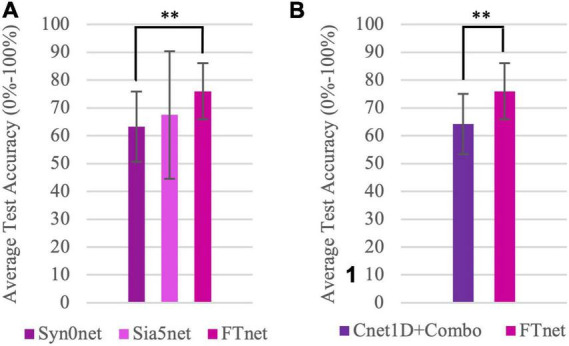
Task-Transfer Experiments results. **(A)** Comparison of classification accuracy of combined movements database including 6 movement classes (Chance level = 16.67%) averaged over all Nearlab subjects using the three stages of task-transfer analysis. Syn0net leverages the synergies found in basic movements utilizing no samples from target domain. Sia5net, a few-shot learning method, uses 5 query samples of each class of target domain. FTnet, employs fine-tuning method with few seconds of target domain data. **(B)** Comparison of classification accuracy of Cnet1D network trained only on combined movements dataset (Cnet1D + Combo) and FTnet framework which leverages the information learned from basic hand movements (source domain), when tested on combined movement dataset (chance level = 16.67%) and averaged over all subjects. This panel highlights the effect of pre-training on source domain. **Corresponds to *p* < 0.01.

Figure 7A displays the experimental results for stage 1, 2, and 3. In Syn0net (stage 1), Sia5net (stage 2), and FTnet (stage 3), zero, few samples and few seconds of labeled target domain data is used for training, respectively and this panel shows this transition. In Syn0net, classification accuracy of combined movements (combination of a hand function and a wrist rotation) is reported for the Nearlab database when Cnet1D was trained only with data of basic movements. Further investigation revealed that most of the classification errors were associated with detecting hand function rather than detecting its wrist rotation (having average hand function classification accuracy of 71.51% and average rotation classification accuracy of 90.24% among Nearlab subjects). It is important to mention that, having Softmax as the final layer activation function may penalize the prediction uncertainties between classes (through increasing loss value). At Syn0net, this is not an issue since the training data only contains basic movements (for which the network should not produce any doubts). Nevertheless, a new set of classifiers with Sigmoid as their final layer activation function, were trained and tested with the same procedure. The results revealed similar performance having 6-movement average accuracy of 63.27%. Classification accuracy of Sia5net using the proposed few-shot learning Siamese network revealed a noteworthy improvement in the performance, yielding 67.19% average accuracy across Nearlab subjects. Average accuracy for FTnet was 75.97% showing more than 10% improvement in accuracy when compared with Syn0net, using only around 430 samples (approximately 5 s of movement) from target domain (combination movements). This improvement was statistically significant with *p* = 0.0097.

Figure 7B is provided as a response to the following question: Assuming there is shared information between basic and combined movements, does knowledge learned from basic movement improve the performance of a classifier trained on the combined movement? To answer this question, the best performing classifier (Cnet1D) is trained using the one repetition of combined movement dataset, referred to as Cnet1D + Combo. The classification accuracy of this network was compared with FTnet. As a reminder, FTnet was pre-trained with basic movement data and fine-tuned with one repetition of combined movement data (same repetition used for training Cnet1D + Combo). Hence comparing Cnet1D + Combo and FTnet shows the effect of pre-training on source domain. The Cnet1D + Combo, Cnet1D network trained only with target domain data, yielded 64.21% average accuracy. In the Figure 7B a 10% improvement in accuracy is observed when the information learned from basic movement dataset is transferred using the framework discussed in stage 3. This improvement was statistically significant with *p* = 0.0097.

## Discussion

The study aimed to apply transfer learning approaches for hand movement intention detection based on sEMG signals as a solution for training deep learning algorithms with limited EMG data. These algorithms can be used in EMG-controlled devices for human-computer interaction, with possible repercussions on hand prosthesis. We proposed two deep learning architectures as the base classifiers and two transfer learning algorithms with two different perspectives, taking advantage of similarities found in EMG patterns of different subjects or different tasks.

The following discussed results are related to Nearlab and Ninapro DB2 databases. The number of movement classes for Nearlab, and Ninapro databases are 8 and 17, respectively. Also, the number of repetitions per movement class is higher in Nearlab comparing to Ninapro DB2. Consequently, the average accuracy of Nearlab is higher than Ninapro DB2 in all experiments. Another notable difference between the two databases is the addition of combined movements in the Nearlab dataset.

The proposed deep network approaches can perform equally or more accurate comparing to reference classical classifiers for both tested databases (Figures 5A, 6A). We have shown that when subject-transfer learning is applied to these deep networks, they can achieve the highest accuracy comparing to classical and deep learning approaches (Tables 4, 5). Hence, it can be concluded that other subjects’ data can be used to enhance the performance of the deep network for the target subject. Figures 5B, 6B exhibit the particular application of transfer learning when the available data for training is limited for Nearlab and Ninapro, respectively. These figures show that in any case of training data size, the PFCnet improves the classification accuracy. Wilcoxon pairwise ranked test also reveals the statistically significant difference between the deep network and PFCnet. It can be concluded that generally, the improvement induced by PFCnet is more when the dataset size is less.

Based on Figures 5C, 6C, it can be inferred that the proposed transfer learning approach can be beneficial to enhance the classification accuracy in all subjects of the Nearlab database and Ninapro DB2. In the Nearlab dataset, subject 10 has the biggest improvement of 4.02% when applying TL to Cnet2D, and subject 2 has the biggest improvement of 2.80% when applying TL to Cnet1D. The biggest improvement for Ninapro subjects is for subject 14, with an 8.89% improvement, and the lowest improvement is 0.83% related to subject 34, according to Figure 5C.

Figure 6C shows the performance of PFCnet in Nearlab dataset, when trained with increasing number of source-subjects. In order to be able to draw more general conclusions, the experiment is repeated with different training data sizes. All trends indicate that using up to 8 subjects can lead to visible improvement of classification performance. Whereas, higher number of source-subjects do not reveal significant improvement in performance of the proposed subject-transfer framework.

Between the two proposed CNN networks, Cnet1D for the Nearlab dataset and Cnet2D for the Ninapro dataset achieved the higher average accuracy. Cnet2D classifier is exploiting the relationship among adjacent channels in the feature extraction part. Since the electrode placement is different in Ninapro and Nearlab datasets, Cnet2D is expected to behave differently.

In 2016, Zhai et al. (2016) achieved 75.74% average accuracy over Ninapro database 2 exercise B. To extract features from EMG data, they converted raw EMG to spectrograms and then applied principal component analysis; finally, they fed spectrograms to SVM. Later in 2017 (Zhai et al., 2017), they improved their results to 81.07%. They also proposed a CNN classifier which obtained 82.22% average accuracy for 17 movements of exercise B. Moreover, they implemented a self-recalibrating method, which was trained on a single repetition (as opposed to 4 repetitions, which was used in their previous reported accuracies); thus, this result is not comparable with the results of this study. Another recent work (Huang and Chen, 2019), which have proposed a hybrid classifier and again have used spectrogram representations of sEMG signals as the input of their classifiers, reported 80.929% for average accuracy over DB2 subjects for exercise B. A summary of state-of-the-art results is presented in Table 6. As it can be observed, our proposed deep network augmented with subject-transfer learning achieves the average accuracy of 82.87% for Ninapro DB2 subjects exercise B, which is higher than the presented results. In contrast to the literature, the input of our classifier is raw EMG data; hence no steps prior to giving the data to the classifier are required.

**TABLE 6:** Comparison of performance of proposed methods with state-of-the-art on Ninapro DB2 database with 17 movement classes (chance accuracy level = 5.88%).

Method	Classification accuracy
SVM + spectrogram + PCA (Zhai et al., 2016)	75.74%
SVM + spectrogram + PCA (Zhai et al., 2017)	81.07%
CNN + spectrogram + PCA (Zhai et al., 2017)	82.22%
CNN-LSTM + spectrogram (Huang and Chen, 2019)	80.929%
**Cnet2D + raw signal**	**81.43%**
**PFCnet + raw signal**	**82.87%**

Bold values represent results obtained with the proposed approaches.

The idea of leveraging the other subjects’ sEMG data has been previously studied (Cote-Allard et al., 2019; Kim et al., 2020; Hoshino et al., 2022). Cote-Allard et al. (2019) have tested their proposed transfer learning method on a self-developed database and Ninapro DB5. They suggested three different data representations to be used with their classifiers: raw, spectrogram, and continuous wavelet transform (CWT), from which CWT representation had the best performance. The improvement gained by their subject-transfer learning method on Ninapro DB5 when trained with four first trials and tested on two remaining trials using CWT was 3.68%, and using raw data was 2.66%. In comparison, we have improved the classification accuracy of Ninapro DB2 using raw data by 1.44% when trained with four trials and tested with two. It should be noted that the range of accuracies differs in the two databases (60–70% in the case of DB5 and 75–85% in the case of DB2). Kim et al. (2020) have tested their CNN-based transfer learning method on Ninapro DB2 all 50 movements, hence the direct comparison of results is not possible. The improvement in accuracy when their network was trained only with the first repetition of data is 2.76%, while our proposed method improved the result of Cnet2D by 5.48%. However, their network was tested on the five remaining repetitions, but our network was tested only on repetitions 2 and 5. From reported results, it can be inferred that the proposed PFCnet subject-transfer framework can increase the performance of a deep classifier using other subjects’ data. Moreover, authors of Hoshino et al. (2022) have compared application of transfer learning on classical classifiers and deep neural networks, reporting that combination of transfer learning with deep neural network achieves highest accuracy. This confirms our results, supporting the move from feature engineering to feature learning.

The novelty of this study in comparison with the mentioned TL studies, in addition to a new subject-transfer framework, is exploring and designing/testing new mechanisms for task-transferability in different movement groups. A further element is the inclusion of the sEMG variability introduced by different hand orientations in the created dataset. An additional consideration, which makes the results of this paper closer to hardware implementation, is its consideration on execution time in accordance with real-time criteria.

According to Figure 7A Syn0net is able to achieve an average accuracy of 63.27% when trained with 8 basic movements and tested on 6 combined movements. Such a procedure (stage 1 of task-transfer framework) could be used to increase the number of output movement classes without the need to increase the size of the training database. The same approach might be exploited to improve EMG-based personalized devices setting in both assistance and rehabilitation fields (Gandolla et al., 2018). This investigation reveals that there is valuable shared knowledge between the two movement groups (basic and combined movement groups) and muscle synergies can be taken into account when designing frameworks to transfer the information between the two movement groups. Furthermore, few-shot learning (as implemented in Sia5net) is proven to be a viable solution for adding new movement classes using as few as 5 new samples per class. The use of 5 query samples from new classes have shown to increase the accuracy of the classifier about 4% when compared to Syn0net. The advantage of such method is that it is applicable to any set of source and target hand movements. Meanwhile, this approach provides an opportunity for the user prioritize distinguishing desired movement classes above others. This feature becomes specifically relevant when combined movements are considered, since we don’t want the two classes that are combined to be distinguished by the classifier. Finally, employing FTnet, it has been shown that using few seconds of movement for each new class (around 1 repetition) the performance of the classifier can be significantly improved when compared to the first two stages.

Figure 7B provides a direct comparison between a classifier only trained on the target domain data and another classifier using the same target domain data alongside information learned from source domain. The statistically significant improvement proves the applicability and effectiveness of our proposed FTnet. Thus, it can be concluded that not only there is shared information between the basic and combined hand movements, but this information can be translated effectively to improve the classification performance on the combined movements.

Task-transfer learning has been previously examined by Cote-Allard et al. (2019), Chen et al. (2021), and Rahimian et al. (2021). The proposed method of Cote-Allard et al. (2019) have improved the classification accuracy of their target domain movement classes (11 classes) from 46.06 to 49.41% using a source domain with 18 movement classes. Authors of Chen et al. (2021) performed task-transfer on two HD-EMG databases from different subjects, the training dataset had 30 gestures and testing dataset included 10 different gestures. They showed that with one repetition of training data their proposed TL algorithm can increase the classification accuracy from 55.63 to 93.32%. This amount of increase is explained by the variety of source gesture set (30) and the number of electrodes (128). However, none of these studies considered the possibility of existing common patterns between basic and combined movements. Moreover, this study confirms results reported by Rahimian et al. (2021) regarding effectiveness of few-shot learning in task-transfer learning. Additionally, this study has provided a specific application of this method for classifying target domain classes that could be a combination of source domain classes. This work’s main limitations were the absence of experiments on end-users (amputees or subjects with movement disorders) and multi-day analysis. In the former case, the most anticipated challenge is the electrode placement due to the fact that the available muscles for EMG acquisition are limited and could be different from subject to subject. In the case of multi-day analysis, variations in electrode positioning, and skin impedance in different sessions could affect the classification’s robustness.

## Conclusion and future work

In this paper, two methods of transfer learning for hand movement classification were investigated. A subject-transfer framework was proposed, which took advantage of shared information among all subject’s sEMG data. A CNN-based network was pre-trained on the target subject while another network was pre-trained on all subjects except the target subject. Feature extractor parts of these two networks were frozen and concatenated at the final layer. Finally, a classifier part was added to complete the final network. The subject-transfer framework was tested on the Ninapro DB2 dataset and a dataset developed on purpose for this paper. Experimental results proved that the proposed framework could enhance the classification accuracy using other subject data and act as a feasible solution when the target subject’s data is not sufficient.

Moreover, three task-transfer frameworks were proposed to classify combined hand movements in 3 stages. Initially, the network trained only with basic movement’s sEMG data (Syn0net) was used to classify combined movements. In the later stages, task-transfer mechanisms including few-shot learning (Sia5net) and fine-tuning using minimum training data from target domain (FTnet) were implemented to classify the combined hand movements. According to experimental results, these frameworks were able to transfer knowledge from basic tasks to combined tasks significantly increasing classification accuracy with minimum training data for combined movements. The proposed method could be beneficial when the aim is to increase the number of movement classes without acquiring a big training dataset. Therefore, these three frameworks could be employed to avoid acquiring large databases from each individual.

In the future, the performance of proposed methods will be further evaluated using databases containing multi-session recordings and end users. Moreover, the application of the proposed frameworks will be investigated in online experiments. The pre-processing steps and classification method will be modified to meet the conditions of real-time classifications, and the effect of visual feedback will be investigated. Additionally, the effect of cross-subject variability in our database can be further investigated with the methods inspired from recent literature (Zancanaro et al., 2021).

## Data availability statement

The datasets presented in this study can be found in online repositories. The names of the repository/repositories and accession number(s) can be found below: github.com/Rahil-Soroush/Nearlab_sEMG_dataset.

## Ethics statement

The studies involving human participants were reviewed and approved by the Ethical Board of Politecnico di Milano (Opinion no. 26/2019). The patients/participants provided their written informed consent to participate in this study.

## Author contributions

RS and SJ contributed to conceive the study, and experimental protocol definition, acquired, analyzed the data, and drafted the manuscript. MG and AP conceived the study, contributed to the experimental protocol definition, performed data interpretation, supervised the project, and substantially contributed to manuscript writing. All authors read and approved the final version of the manuscript.
